# The effectiveness of non-invasive vagus nerve stimulation in migraine management: a meta-analysis of randomized controlled trials

**DOI:** 10.3389/fneur.2026.1842386

**Published:** 2026-07-27

**Authors:** Chenjie Qian, Yuebinquan Shi, Chunhua Liu, Zegen Ye, Yue Jin, Huaqiang Wang, Di Li

**Affiliations:** 1 The People’s Hospital of Cangnan, Wenzhou, Zhejiang, China; 2 Lishui Hospital of Traditional Chinese Medicine Affiliated to Zhejiang University of Chinese Medicine, Lishui, Zhejiang, China

**Keywords:** headache, meta-analysis, migraine, non-invasive vagus nerve stimulation (nVNS), vagus-nerve stimulation

## Abstract

**Background:**

Non-invasive vagus nerve stimulation (nVNS) has emerged as a potential non-pharmacological intervention for migraine. This systematic review and meta-analysis of randomized controlled trials (RCTs) evaluated its efficacy for both acute and preventive migraine treatment.

**Methods:**

This review was conducted according to PRISMA and registered in PROSPERO (CRD420261347799). PubMed, Cochrane Central, Embase, CNKI, VIP, and Wanfang were searched from inception to February 19, 2026, to identify RCTs of nVNS for migraine. The primary outcomes were monthly headache days, monthly acute medication use, and headache pain intensity. Continuous outcomes were synthesized as mean differences (MDs), and dichotomous outcomes as odds ratios (ORs), with 95% confidence intervals (CIs). Fixed- or random-effects models were applied according to outcome type and statistical heterogeneity, and random-effects sensitivity analyses were conducted for continuous primary outcomes to account for expected clinical heterogeneity. Risk of bias was assessed using the original Cochrane Risk of Bias tool, and certainty of evidence was evaluated using GRADE.

**Results:**

Seven RCTs involving 917 patients with migraine were included. Meta-analysis showed that nVNS significantly reduced monthly headache days (MD = −1.52, 95% CI − 1.86 to −1.18, *p* < 0.001), monthly acute medication days (MD = −1.18, 95% CI − 1.72 to −0.63, *p* < 0.001), and headache pain intensity (MD = −0.97, 95% CI − 1.30 to −0.64, *p* < 0.001), with low heterogeneity across these outcomes. Exploratory overall estimates for acute treatment outcomes also favored nVNS for pain-free and pain-relief responses within 2 h, although these findings were interpreted cautiously because repeated time-point data from the same trials may be correlated. Overall risk of bias was low. GRADE certainty was high for monthly headache days and monthly acute medication days, and moderate for headache pain intensity, acute treatment outcomes, and adverse events.

**Conclusion:**

nVNS may reduce monthly headache days, acute medication use, and headache pain intensity, while also improving acute pain-free and pain-relief responses within 2 h in patients with migraine. It may represent a promising non-pharmacological option for migraine treatment and prevention, although further high-quality studies are needed to confirm its long-term efficacy and safety.

**Systematic review registration:**

https://www.crd.york.ac.uk/prospero/display_record.php?ID=CRD420261347799, identifier: CRD420261347799.

## 1 Introduction

Migraine is a common primary headache disorder with substantial disability burden, usually manifested by recurrent moderate-to-severe throbbing headaches lasting 4–72 h, together with symptoms such as nausea, vomiting, and sensitivity to light and sound (1). Migraines significantly disrupt daily life, work capacity, and social participation. As a highly prevalent neurological disorder worldwide, it creates a considerable burden on individuals, families, and healthcare systems (2). While several medications are available for acute treatment and prevention, their effectiveness varies across individuals, and many patients do not experience optimal symptom control (3, 4). Moreover, long-term use of these drugs can lead to side effects such as dizziness, fatigue, gastrointestinal issues, and medication-overuse headaches, which can limit their clinical applicability (5, 6). Consequently, finding safe, effective, and well-tolerated non-drug treatment options is essential for improving migraine management.

The vagus nerve, a major branch of the parasympathetic nervous system, plays an essential role in regulating multiple physiological activities, including breathing, heart rate, gastrointestinal function, and inflammatory responses (7, 8). Because it is composed mainly of afferent fibers, it serves as an important link between peripheral input and central pain-related structures, including the nucleus tractus solitarius, spinal trigeminal nucleus, locus coeruleus, thalamus, and hypothalamus (9). These anatomical and functional connections provide a rationale for the use of vagus nerve stimulation in migraine, as it may influence trigeminovascular activity, central pain pathways, and neuroinflammation (10). Compared with implantable devices, non-invasive vagus nerve stimulation (nVNS) offers a simpler and more acceptable approach because it avoids surgical implantation. Depending on the stimulation site, nVNS can be applied to the cervical or auricular branch of the vagus nerve, and both have been investigated as potential treatments for migraine.

In recent years, nVNS has gained increasing attention as a potential option for both acute and preventive migraine management. Several randomized controlled trials (RCTs) have suggested that nVNS may improve pain relief, reduce acute medication use, and lower headache frequency (11, 12). However, findings across studies have not been fully consistent, with some trials showing no significant effect on migraine pain intensity (13). These discrepancies may be related to variations in stimulation sites, stimulation parameters, treatment duration, and outcome measures among studies. In addition, previous systematic reviews and meta-analyses have included only a limited number of RCTs and have mainly focused on pain relief or short-term outcomes, leaving other clinically relevant endpoints, such as headache days, acute medication-use days, and pain intensity, insufficiently evaluated (14, 15). Therefore, this systematic review and meta-analysis incorporated more recent RCTs to provide a more comprehensive evaluation of the efficacy of nVNS for migraine treatment and prevention, intending to offer stronger evidence for clinical decision-making.

## 2 Methods

The present systematic review and meta-analysis were conducted in accordance with the PRISMA statement and prospectively registered with PROSPERO under registration number CRD420261347799. The registration record is available at: https://www.crd.york.ac.uk/PROSPERO/view/CRD420261347799.

### 2.1 Search strategy

This review consisted of three main stages: initial identification of records, multistage screening, and final study inclusion. A comprehensive search was conducted to identify RCTs published from database inception to February 19, 2026, in PubMed, Embase, the Cochrane Library (CENTRAL), CNKI, Wanfang Data, and VIP Database. No language restrictions were applied, and studies published in any language, including English and Chinese, were considered eligible for screening.

The search strategy was developed around three core components: terms related to the condition, the intervention, and the study design. Migraine-related terms included different expressions and subtypes of migraine, such as “migraine,” “migraine disorder,” “migraine headache,” “abdominal migraine,” “hemicrania migraine,” and “cervical migraine syndrome.” Intervention-related terms included “vagus nerve stimulation,” “vagal nerve stimulation,” and their variants. Terms identifying RCTs were also incorporated. Both controlled vocabulary (e.g., MeSH terms) and free-text keywords were used, and truncation symbols (*) were applied where appropriate to capture variations in terminology. Reference lists of the included studies were also manually checked for potentially missed records, and authors of conference abstracts were contacted, when possible, for additional information. The complete search strategy is shown in Supplementary Table 1.

### 2.2 Inclusion and exclusion criteria

Study selection was based on the PICOS framework, which addressed population, intervention, comparator, outcomes, and study design.

Studies were included if they met the following criteria:

1. Population: patients diagnosed with migraine according to the International Classification of Headache Disorders (ICHD) criteria reported in the original trials, including ICHD-IIR, ICHD-2, and ICHD-3, without other primary or secondary headache disorders.
2. Intervention: nVNS, including cervical nVNS or auricular nVNS/taVNS.
3. Comparator: sham stimulation, usual care, conventional treatment, or other non-active control conditions.
4. Outcomes: studies reporting at least one efficacy or safety outcome relevant to migraine management. For preventive or repeated-treatment trials, eligible outcomes included monthly headache days, monthly acute migraine medication days, headache pain intensity, or other outcomes reported after a defined treatment or follow-up period, generally 4 weeks or longer. For acute-attack trials, eligible outcomes included post-treatment responses after treated migraine attacks, such as pain-free or pain-relief responses within 2 h, regardless of whether the acute assessment was embedded in a longer double-blind study period.
5. Study design: randomized controlled trials.

Exclusion criteria were as follows:

1. non-original publications, such as letters, case reports, conference abstracts, reviews, or comments;
2. studies without sufficient data to calculate effect estimates and their 95% confidence intervals;
3. trials that did not report efficacy or safety outcomes; and
4. studies with duplicate publications or overlapping datasets.

### 2.3 Study selection

Two reviewers (C.Q. and C.L.) independently screened studies based on the predefined inclusion and exclusion criteria. Retrieved records were managed in EndNote (Clarivate Analytics), where duplicates were removed. Titles and abstracts were first reviewed to exclude irrelevant studies, and the full texts of potentially eligible articles were then assessed for final inclusion. Disagreements were settled by discussion or, when needed, by consultation with a third reviewer (H.W.). When multiple publications reported data from the same trial population, they were treated as reports of a single independent RCT. The main trial report was used for study characteristics and participant information, whereas secondary or *post hoc* reports were used only as supplementary sources for outcome extraction when relevant data were not fully available in the main report. Participants from the same trial population were counted only once in the overall study count and sample size summary.

### 2.4 Data collection

A standardized data extraction form was used to collect relevant information from the included studies. Extracted data included the first author, year of publication, country, study design, sample size, participant characteristics, follow-up duration or post-treatment assessment time points, outcome measures, and adverse events. Stimulation type was classified according to the reported stimulation site as cervical nVNS or auricular nVNS/taVNS. Detailed information on the intervention and control conditions was also recorded, including the type of intervention, stimulation device, stimulation site, stimulation parameters, treatment frequency, duration of each session, total treatment period, acute post-treatment assessment window, and comparator regimen, to capture variations in treatment protocols across studies. The primary outcomes were monthly headache days, monthly acute migraine medication days, and pain intensity, while the secondary outcomes were pain-free and pain-relief responses within 2 h after treatment. For outcomes reported at multiple assessment time points, the corresponding time points were extracted separately. When multiple publications reported data from the same trial population, the main trial report was used as the primary source for study characteristics and participant information, whereas secondary or *post hoc* reports were used only as supplementary sources for extracting additional outcome data not fully available in the main report. Data extraction was performed independently by two reviewers (C.Q. and C.L.), and any disagreements were resolved through discussion or, when necessary, by consultation with a third reviewer (H.W.).

### 2.5 Assessment of risk of bias

The risk of bias of the included independent RCTs was assessed using the original Cochrane Risk of Bias tool. This tool evaluates bias across the following domains: random sequence generation, allocation concealment, blinding of participants and personnel, blinding of outcome assessment, incomplete outcome data, selective reporting, and other potential sources of bias. Each domain was judged as having a low, unclear, or high risk of bias, and an overall risk-of-bias judgment was made for each trial. For multiple reports from the same trial population, risk of bias was assessed at the trial level, based primarily on the main trial report and supplemented by related secondary or *post hoc* reports when necessary.

As non-pharmacological interventions such as neuromodulation are particularly prone to performance and detection bias, blinding procedures and control conditions were evaluated with special attention. This included blinding of participants, study personnel, and outcome assessors, as well as the use of sham stimulation or usual care as comparators. Differences in blinding approaches and control design across studies were documented and taken into account when interpreting the pooled results. Two reviewers (C.Q. and Z.Y.) independently completed and cross-checked all assessments, and any discrepancies were resolved by a third reviewer (H.W.).

### 2.6 Subgroup analysis

To explore potential clinical heterogeneity by stimulation modality, exploratory subgroup analyses were conducted by stimulation type for the primary outcomes: monthly headache days, monthly acute migraine medication days, and headache intensity. Stimulation type was classified according to the reported stimulation site as cervical nVNS or auricular nVNS/taVNS. Because the number of independent trials and effect estimates within each subgroup was limited, these subgroup analyses were considered exploratory and were not intended to provide definitive evidence of differential efficacy between stimulation modalities. Therefore, between-subgroup comparisons were interpreted cautiously.

### 2.7 Sensitivity analysis

Sensitivity analyses were performed for the primary outcomes using a leave-one-out approach. In this process, each included study was sequentially removed, and the pooled effect estimate was recalculated to evaluate the influence of individual studies on the overall results. In addition, given the expected clinical heterogeneity across studies, supplementary random-effects sensitivity analyses were conducted for the continuous primary outcomes, including monthly headache days, monthly acute migraine medication days, and headache intensity. These analyses were used to examine whether the main findings remained robust when between-study variability was considered.

### 2.8 Statistical analysis

Data were analyzed using RevMan 5.4. Effect sizes were calculated from the sample size and post-intervention mean ± standard deviation values of both groups. For continuous outcomes assessed on the same scale, pooled estimates were expressed as mean differences (MDs) with 95% confidence intervals (CIs). For the main analysis of continuous outcomes, a fixed-effect model was used when statistical heterogeneity was not substantial (*I*^2^ < 50%), whereas a random-effects model was used when substantial heterogeneity was detected (*I*^2^ ≥ 50%). Given the expected clinical heterogeneity across studies, including differences in stimulation type, stimulation parameters, treatment duration, treatment intent, migraine subtype, and control conditions, additional random-effects sensitivity analyses were performed for the continuous primary outcomes. Dichotomous outcomes, including pain-free responses, pain-relief responses, and adverse events, were synthesized as odds ratios (ORs) with 95% CIs using a random-effects model. Heterogeneity was assessed using Cochran’s *Q* test and the *I*^2^ statistic. Forest plots were generated for all analyses, and a two-sided *p*-value ≤ 0.05 was considered statistically significant.

For acute treatment outcomes reported at multiple post-treatment assessment time points, data were stratified according to the corresponding time points. Pooled estimates were calculated within each time-point subgroup to clarify time-specific treatment effects. In addition, an exploratory overall estimate across time points was retained to summarize the general direction of the treatment effect. Because repeated time-point estimates from the same trial population may be statistically correlated and may not represent fully independent observations, the exploratory overall estimate was interpreted cautiously and was not considered the primary estimate for time-specific effects. Therefore, subgroup-specific results were prioritized when interpreting acute treatment outcomes.

Given that fewer than 10 independent RCTs were included for each outcome, formal evaluation of publication bias, including funnel plot analysis and statistical testing, was not undertaken. This decision was consistent with the Cochrane Handbook, which advises that such methods are of limited reliability when fewer than 10 studies are included (16).

### 2.9 Certainty of evidence assessment

The certainty of evidence was evaluated for all pooled outcomes using the GRADE framework, covering primary continuous outcomes, acute treatment outcomes, and adverse events. Five domains were considered: risk of bias, inconsistency, indirectness, imprecision, and publication bias. The certainty level was classified as high, moderate, low, or very low. Imprecision was assessed by considering the number of studies, total sample size or event numbers, and the width and clinical relevance of the 95% CI. Outcomes were not downgraded solely because of sample size when the confidence interval was relatively narrow, did not cross the no-effect line, and the effect direction was consistent.

## 3 Results

### 3.1 Study selection process

**Figure 1:**
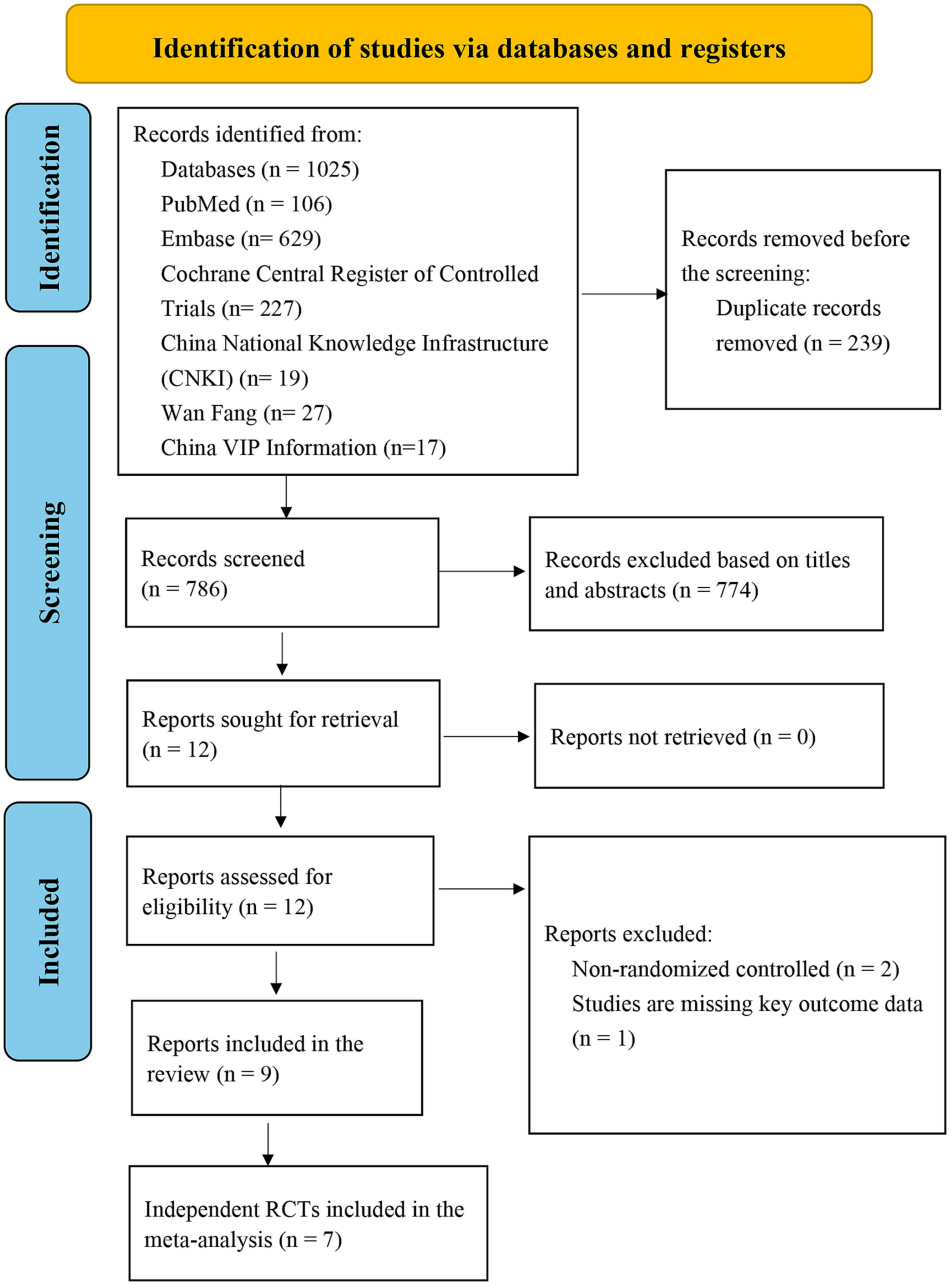
Flow diagram of the literature search and study selection based on PRISMA guidelines.

Figure 1 presents the study selection flow. Overall, 1,025 records were identified from databases and trial registries. After 239 duplicates were removed, 786 records underwent title and abstract screening; 774 were excluded. The remaining 12 articles were reviewed in full text. After full-text evaluation and reference checking, three studies were excluded because two were non-randomized and one was a conference abstract. Ultimately, nine publications corresponding to seven independent RCTs were eligible for inclusion in the review. Among these, Tassorelli et al. (21), Grazzi et al. (11), and Martelletti et al. (2018) were multiple reports from the same PRESTO trial population and were therefore treated as one independent RCT.

### 3.2 Characteristics of the included studies

A total of seven independent RCTs involving 917 unique participants were included in this review, with publication years ranging from 2015 to 2025. The sample sizes of the independent trials ranged from 44 to 332 participants. Overall, 458 patients received nVNS in the intervention groups, while 459 participants in the control groups received usual care or sham stimulation. The proportion of female participants varied from 37.5 to 87.5% across studies.

Regarding intervention type, four studies evaluated cervical nVNS, and three evaluated auricular nVNS/taVNS. The included trials covered both acute treatment and preventive management settings, with protocols ranging from single-attack acute treatment with short-term post-treatment assessment to repeated stimulation over 4–12 weeks. Reported stimulation frequencies or burst repetition rates ranged from 1 to 25 Hz, depending on the stimulation modality and device. Six independent trials provided information on nVNS-related adverse events. The included trials were conducted in China (*n* = 3, 17–19), Germany (*n* = 2, 13, 20), the United States (*n* = 1, 12), and Italy (*n* = 1, 21). The main characteristics of the included studies are summarized in Table 1.

**Table 1:** Overview of the Included Studies.

**Study**	**Country**	**Design**	**nVNS group**								**Control group**				**Adverse event**	**Outcomes**	**Assessment time point**
			**Sample size**	**Age (year)**	**Female** **(%)**	**Site of stimulation/** **Stimulation type**	**Device/stimulation** **device**	**Stimulation** **parameters**	**Treatment duration**	**Treatment** **Regimen/purpose**	**Sample** **size**	**Age (year)**	**Female** **(%)**	**Intervention**			
Qu and Shao (1)	China	RCT, 2 arms	22	38.2 ± 7.8	13 (59.1%)	Bilateral cervical vagus nerve/cervical nVNS	TENS200A auricular vagus nerve stimulator (Suzhou Medical Supplies Co., Ltd., China)	20 Hz; sparse- dense wave	30 min/session; 2 sessions/day; 5 days/week; 12 weeks	Acute migraine treatment	22	36.2 ± 9.3	12 (54.5%)	Conventional treatment	No AEs were observed.	Number of days with headache, VAS	4, 8, 12 weeks
Sun and Hou (18)	China	RCT, 2 arms	40	45.36 ± 4.16	17 (42.5%)	Auricular concha region/auricular nVNS	BHD-1A transcutaneous electrical stimulation system	20 Hz square-wave pulses; 0.5 mA; stimulation/rest cycle: 30 s/2 min	30 min/session; once daily; 3 months	Acute migraine treatment	40	44.98 ± 4.21	15 (37.5%)	Conventional treatment	NR	Number of days with headache	12 weeks
Diener et al. (13)	Germany	RCT, 2 arms	165	43.5 ± 11.1	142 (86.1%)	Bilateral cervical vagus nerve/cervical nVNS	Active nVNS device	Active: 5 kHz sine wave burst, 1 ms, repeated every 40 ms (25 Hz), 24 V peak, 60 mA peak	Two consecutive Bilateral stimulations per treatment; TID; 12 weeks	Preventive Migraine treatment	167	41.4 ± 12.3	138 (82.6%)	Sham taVNS	Common ADEs included rash, pain, erythema, application-site discomfort, and dizziness; no serious ADEs were reported.	Number of days with headache, Migraine days Acute medication days	12 weeks
Najib et al. (12)	USA	RCT, 2 arms	56	40.3 ± 13.9	49 (87.5%)	Same side of the neck, ipsilateral to the side of predominant pain/cervical nVNS	nVNS device	five 5-kHz pulses repeated at 25 Hz; approximately sine- wave waveform; 24 V peak voltage; 60 mA maximum output current	120 s per stimulation	Preventive treatment; administered 3 times daily	57	44.6 ± 10.7	44 (77.2%)	Sham taVNS	Four SAEs occurred; most AEs were mild.	Number of days with headache, Acute medication days	4 weeks
Straube et al. (15)	Germany	RCT, 2 arms	22	43.8 ± 11.5	18 (81.82%)	Concha of the outer ear (auricular branch of the vagus nerve)/auricular nVNS	NEMOS® t-VNS device (Cerbomed, Erlangen, Germany)	Pulse width: 250 μs; frequency: 1 Hz or 25 Hz; duty cycle:	4 h/day (in 1–4 h sessions)	Daily t-VNS treatment; additional use allowed for acute headache	24	39.3 ± 12.4	21 (87.5%)	Sham taVNS	Most AEs were mild to moderate and mainly involved local stimulation-site reactions; no treatment-related SAEs were reported.	Number of days with headache, Migraine days Acute medication days	12 weeks
PRESTO trial / Tassorelli et al. (21), Grazzi et al. (11) and Martelletti et al. (2018)	Italy	RCT, 2 arms	120	38.8 ± 11.0	95 (79.2%)	Bilateral neck (right and left cervical vagus nerve region)/cervical nVNS	nVNS device	5-kHz sine wave burst lasting 1 ms (5 × 200 μs), repeated every 40 ms (25 Hz), 24 V peak, 60 mA peak	120 s per stimulation	Acute migraine treatment	123	39.6 ± 11.8	91 (74.0%)	Sham taVNS	No SAEs were reported; common AEs included application-site reactions and nasopharyngitis.	Pain relief Pain free	Acute outcomes: 30, 60, and 120 min after treatment; double-blind period: 4 weeks
Zhang et al. (19)	China	RCT, 2 arms	33	NR	NR	Left cymba concha (real taVNS)/auricular nVNS	Electronic acupuncture treatment instrument (SDZ-II, Huatuo, Suzhou, China)	1 Hz; pulse width 0.2 ms	30 min/session; 12 sessions in total over 4 weeks	Acute treatment of migraine	26	NR	NR	Sham taVNS	Not reported	Number of days with headache, Migraine days Acute medication days	4 weeks

RCT: Randomized Controlled Trial; NR: Not Reported; AE: Adverse event; auricular nVNS/taVNS: auricular non-invasive vagus nerve stimulation/transcutaneous auricular vagus nerve stimulation; cervical nVNS: cervical non-invasive vagus nerve stimulation; nVNS: Non-invasive vagus nerve stimulation; ADEs: Adverse Drug Events; SAEs: Serious Adverse Events; VAS: Visual Analog Scale. Tassorelli et al. (21), Grazzi et al. (11), and Martelletti et al. (2018) were multiple reports derived from the same PRESTO trial population. Tassorelli et al. (21) was treated as the main trial report for study design, participant characteristics, and risk-of-bias assessment, whereas Grazzi et al. (11) and Martelletti et al. (2018) were used only as supplementary sources for extracting additional acute treatment outcomes. These PRESTO-related reports were counted as one independent RCT, and participants from the PRESTO cohort were counted only once in the overall sample size.

Of note, Tassorelli et al. (21), Grazzi et al. (11), and Martelletti et al. (2018) were multiple reports derived from the same PRESTO trial population. Therefore, Tassorelli et al. (21) was retained in Table 1 as the main PRESTO trial report, whereas Grazzi et al. (11) and Martelletti et al. (2018) were used only as supplementary sources for extracting acute treatment outcomes and were not counted as separate independent RCTs.

### 3.3 Risk of bias

**Figure 2:**
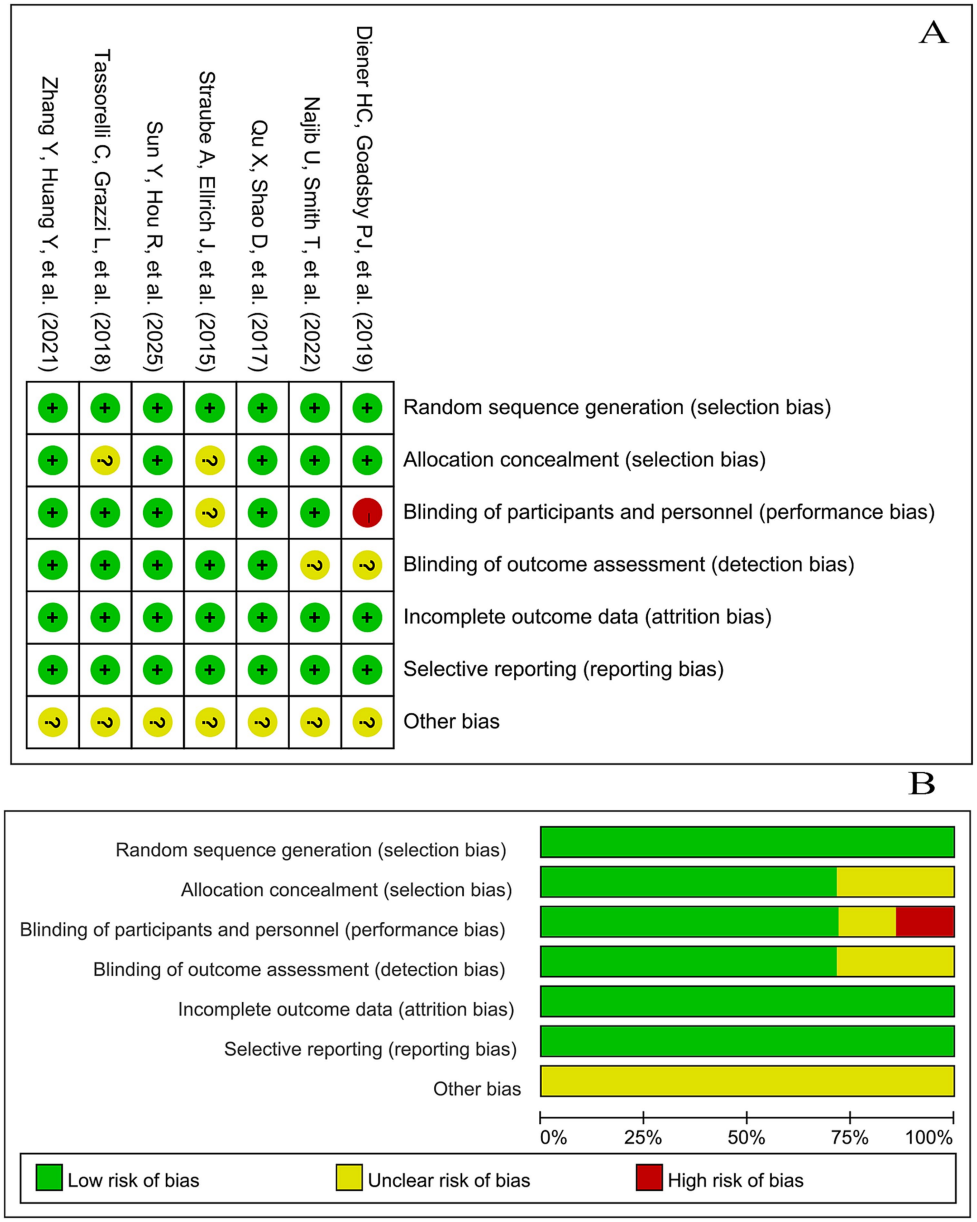
Visualization of the overall risk of bias assessment. **(A)**: Risk of bias summary. “+” means low-risk bias; “?” means unclear risk bias; “–” means high-risk bias; **(B)**: Deviation chart for risk of bias.

The risk of bias of the seven independent RCTs was independently evaluated by two reviewers using the original Cochrane Risk of Bias tool, and the results are shown in Figure 2. For multiple reports from the same trial population, risk of bias was assessed at the trial level, with the PRESTO-related reports treated as one independent trial. Overall, most studies were considered to be at low risk of bias, although several methodological concerns were identified. In one trial, personnel responsible for instructing participants in device use were not blinded, which may have affected participant masking and introduced potential bias. In another trial, endpoint analyses were not based on complete outcome data despite complete trial registration information. Although these issues did not materially change the overall risk-of-bias assessment, they should be considered when interpreting the pooled findings.

### 3.4 Synthesis of results

Our analyses primarily focused on key preventive and burden-related outcomes, including monthly headache days, monthly acute migraine medication days, and headache pain intensity. In addition, this review evaluated acute treatment outcomes, including pain-free and pain-relief responses within 2 h after treatment, as well as safety outcomes. For acute treatment outcomes reported at multiple post-treatment assessment time points, results were further stratified by assessment time point.

#### 3.4.1 Monthly headache days

**Figure 3:**
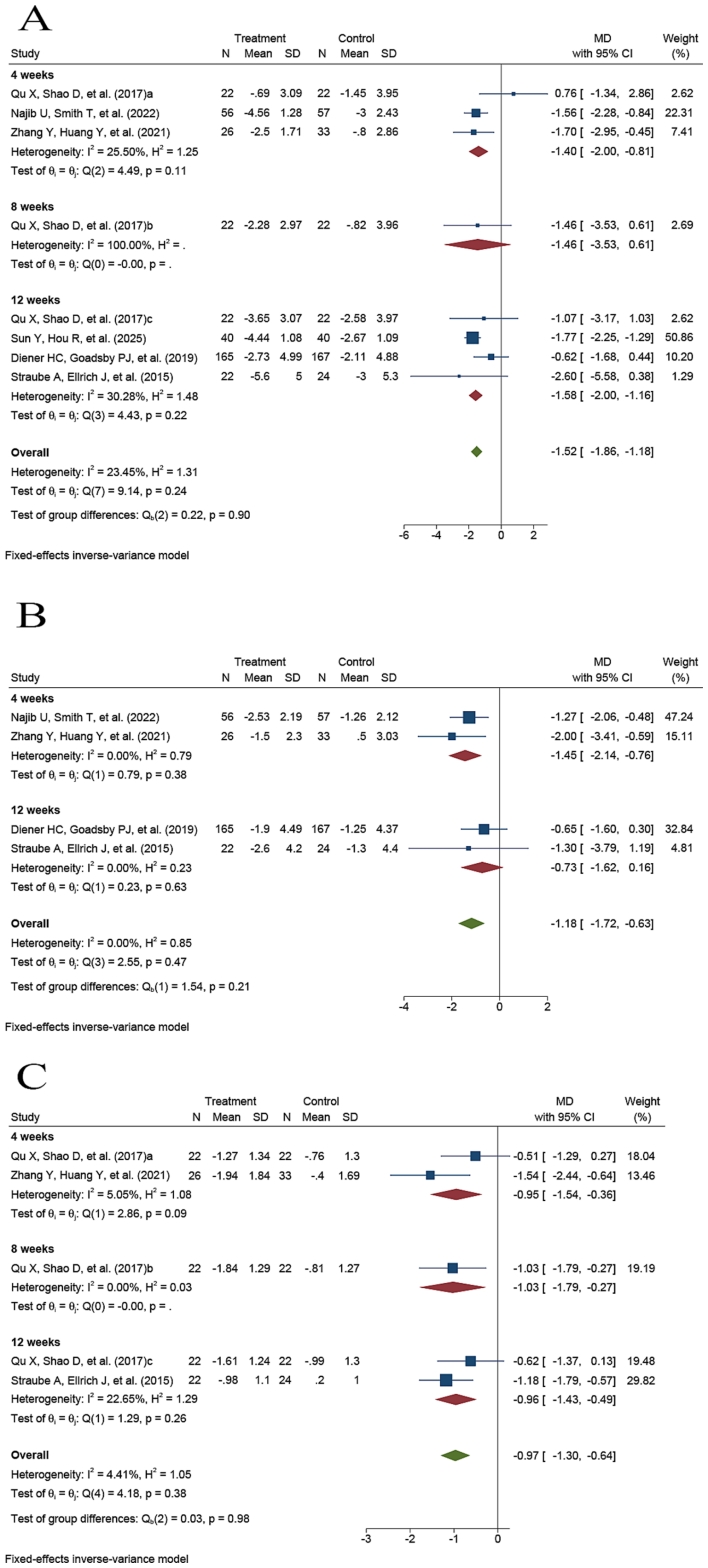
Forest plots illustrating the effects of nVNS on monthly headache days, monthly acute medication days, and headache intensity, stratified by assessment time point. **(A)** monthly headache days; **(B)** monthly acute medication days; **(C)** headache intensity. The overall pooled estimates across time points are exploratory and should be interpreted cautiously because repeated time-point estimates from the same trial population may be statistically correlated.

Six RCTs involving 674 participants reported monthly headache days, contributing eight time-point-specific effect estimates (12, 13, 17–20). The data were stratified according to assessment time point. At 4 weeks, nVNS was associated with a significantly greater reduction in monthly headache days than the control condition (MD = −1.40, 95% CI − 2.00 to −0.81, *p* < 0.001; *I*^2^ = 25.50%). At 8 weeks, the effect estimate also favored nVNS, although the difference was not statistically significant (MD = −1.46, 95% CI − 3.53 to 0.61, *p* = 0.17). At 12 weeks, nVNS showed a significant reduction in monthly headache days (MD = −1.58, 95% CI − 2.00 to −1.16, *p* < 0.001; *I*^2^ = 30.28%). The exploratory overall estimate across time points also favored nVNS (MD = −1.52, 95% CI − 1.86 to −1.18, *p* < 0.001), with low heterogeneity (*I*^2^ = 23.45%, *p* = 0.24) and no significant subgroup difference by assessment time point (*p* = 0.90) (Figure 3A).

#### 3.4.2 Monthly acute medication days

Four RCTs involving 550 participants reported monthly acute migraine medication days, contributing four time-point-specific effect estimates (12, 13, 19, 20). At 4 weeks, nVNS was associated with a significantly greater reduction in monthly acute medication days than the control condition (MD = −1.45, 95% CI − 2.14 to −0.76, *p* < 0.001; *I*^2^ = 0.00%). At 12 weeks, the effect estimate favored nVNS but did not reach statistical significance (MD = −0.73, 95% CI − 1.62 to 0.16, *p* = 0.11; *I*^2^ = 0.00%). The exploratory overall estimate across time points showed a significant reduction in monthly acute medication days with nVNS (MD = −1.18, 95% CI − 1.72 to −0.63, *p* < 0.001), with no observed heterogeneity (*I*^2^ = 0.00%, *p* = 0.47) and no significant subgroup difference by assessment time point (*p* = 0.21) (Figure 3B).

#### 3.4.3 Headache intensity

Three RCTs involving 149 participants reported headache intensity, contributing five time-point-specific effect estimates (17, 19, 20). When stratified by assessment time point, nVNS was associated with a significantly greater reduction in headache intensity at 4 weeks (MD = −0.95, 95% CI − 1.54 to −0.36, *p* = 0.002; *I*^2^ = 5.05%), 8 weeks (MD = −1.03, 95% CI − 1.79 to −0.27, *p* = 0.008), and 12 weeks (MD = −0.96, 95% CI − 1.43 to −0.49, *p* < 0.001; *I*^2^ = 22.50%). The exploratory overall estimate across time points also favored nVNS (MD = −0.97, 95% CI − 1.30 to −0.64, *p* < 0.001), with low heterogeneity (*I*^2^ = 4.41%, *p* = 0.38) and no significant subgroup difference by assessment time point (*p* = 0.98) (Figure 3C).

#### 3.4.4 Pain-free response within 2 h after the first treated attack

**Figure 4:**
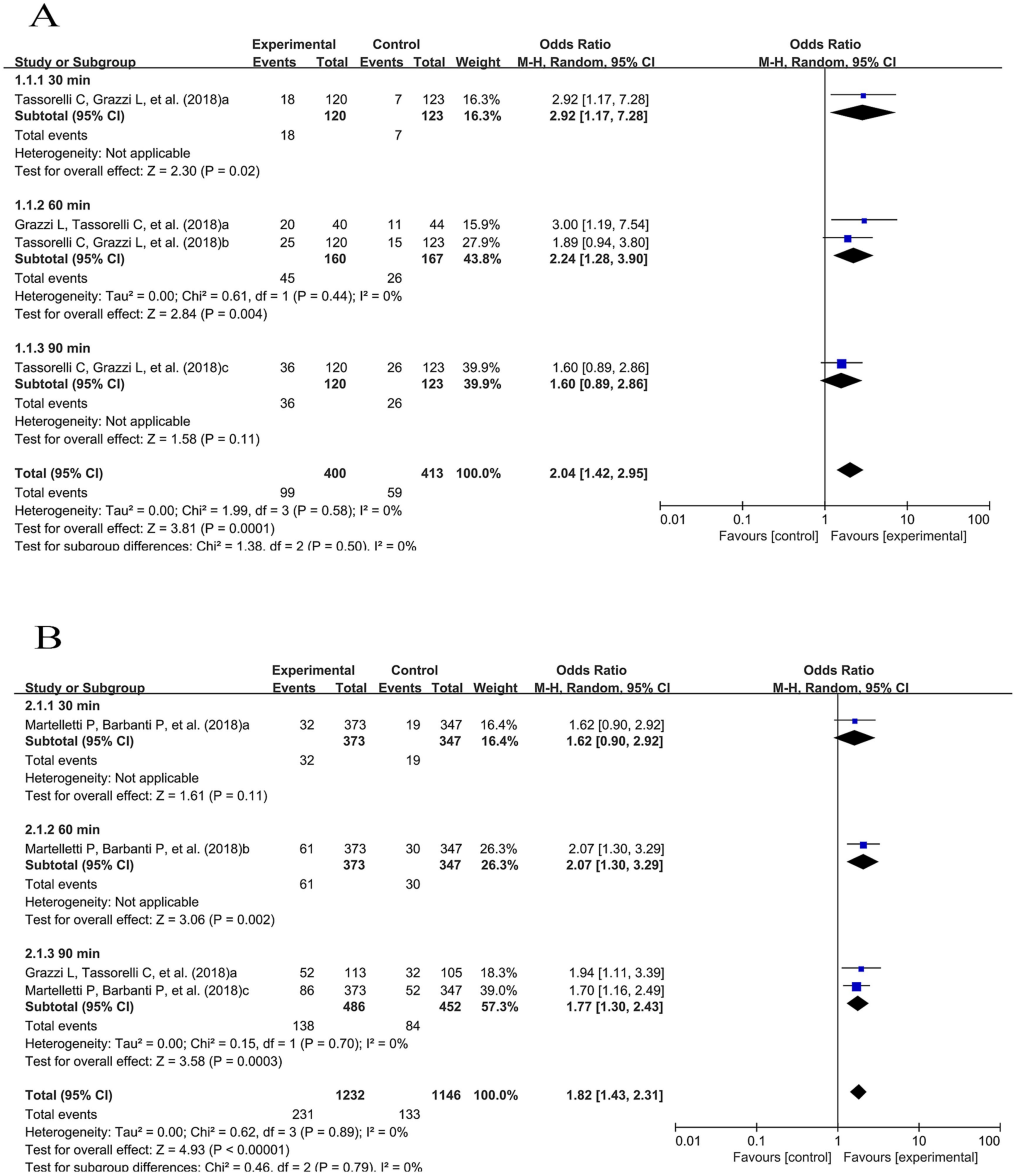
Forest plots of the effects of nVNS on pain-free responses within the 2-h post-treatment period, stratified by post-treatment assessment time point. **(A)** pain-free response within 2 h after the first treated attack; **(B)** pain-free response within 2 h for all treated attacks. The overall pooled estimates across post-treatment time points are exploratory and should be interpreted cautiously because repeated time-point estimates from the same trial population may be statistically correlated.

Two independent RCTs involving 255 participants reported pain-free responses within the 2-h post-treatment period after the first treated attack, contributing four time-point-specific effect estimates (11, 21). These data were stratified according to post-treatment assessment time point. At 30 min and 60 min, the subgroup estimates favored nVNS over the control condition, whereas the effect at 90 min was not statistically significant. The exploratory overall estimate across time points showed that patients receiving nVNS were more likely to achieve pain freedom after the first treated attack than those in the control group (OR = 2.04, 95% CI 1.42 to 2.95, *p* < 0.001). No significant heterogeneity was observed across the included effect estimates (*I*^2^ = 0.00%, *p* = 0.58). Because multiple time-point estimates from the same trial may be statistically correlated, this overall estimate was interpreted cautiously, and the time-point-specific subgroup results were prioritized (Figure 4A).

#### 3.4.5 Pain-free response within 2 h across all treated attacks

Two independent RCTs involving 255 participants reported pain-free responses within the 2-h post-treatment period across all treated attacks, contributing four time-point-specific effect estimates (11, 21). These data were stratified according to post-treatment assessment time point. The subgroup estimates generally favored nVNS over the control condition. The exploratory overall estimate across time points showed that nVNS increased the likelihood of achieving pain freedom across all treated attacks (OR = 1.82, 95% CI 1.43 to 2.31, *p* < 0.001). No significant heterogeneity was observed across the included effect estimates (*I*^2^ = 0.00%, *p* = 0.89). However, because repeated time-point estimates from the same trial may not be statistically independent, this exploratory overall estimate should be interpreted cautiously, with priority given to the time-point-specific subgroup findings (Figure 4B).

#### 3.4.6 Pain-relief response within 2 h after the first treated attack

**Figure 5:**
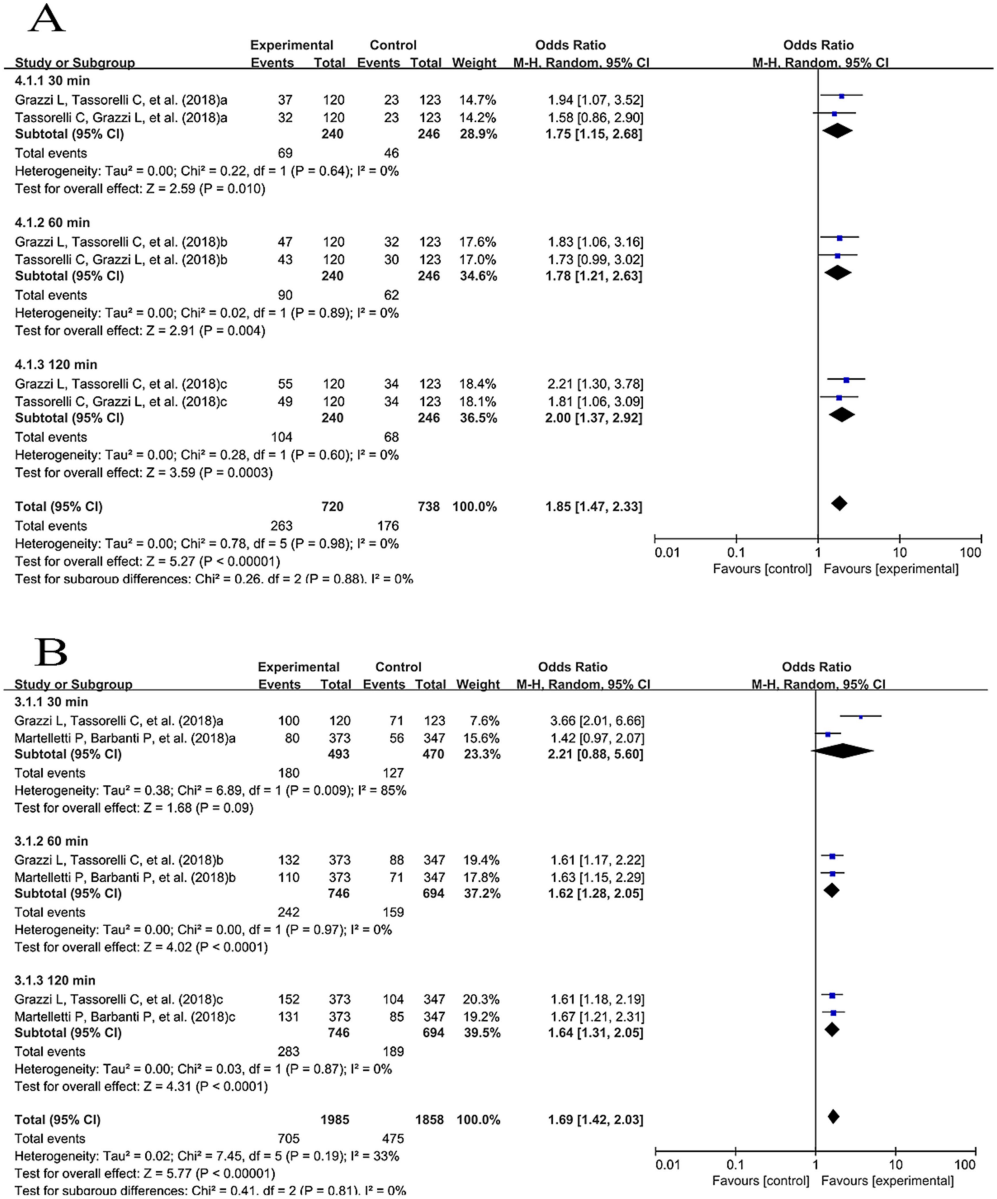
Forest plots of the effects of nVNS on pain-relief responses within the 2-h post-treatment period, stratified by post-treatment assessment time point. **(A)** pain-relief response within 2 h after the first treated attack; **(B)** pain-relief response within 2 h for all treated attacks. The overall pooled estimates across post-treatment time points are exploratory and should be interpreted cautiously because repeated time-point estimates from the same trial population may be statistically correlated.

Two independent RCTs involving 255 participants reported pain-relief responses within the 2-h post-treatment period after the first treated attack, contributing six time-point-specific effect estimates (11, 21). These data were stratified according to post-treatment assessment time point. The exploratory overall estimate across time points showed that, compared with the control group, patients receiving nVNS were more likely to achieve pain relief after the first treated attack (OR = 1.85, 95% CI 1.47 to 2.33, *p* < 0.001). No significant heterogeneity was observed across the included effect estimates (*I*^2^ = 0.00%, *p* = 0.98). Because multiple time-point estimates from the same trial may be statistically correlated, this exploratory overall estimate was interpreted cautiously, and the time-point-specific subgroup findings were prioritized (Figure 5A).

#### 3.4.7 Pain-relief response within 2 h across all treated attacks

Two independent RCTs involving 255 participants reported pain-relief responses within the 2-h post-treatment period across all treated attacks, contributing six time-point-specific effect estimates (11, 21). These data were stratified according to post-treatment assessment time point. The exploratory overall estimate across time points showed that, compared with the control group, patients receiving nVNS were more likely to achieve pain relief across all treated attacks (OR = 1.69, 95% CI 1.42 to 2.03, *p* < 0.001). Low-to-moderate heterogeneity was observed across the included effect estimates (I^2^ = 33.00%, *p* = 0.19), suggesting acceptable consistency of the pooled result. Because repeated time-point estimates from the same trial population may be statistically correlated, this exploratory overall estimate was interpreted cautiously, and the time-point-specific subgroup findings were prioritized (Figure 5B).

### 3.5 Safety and tolerability

**Figure 6:**
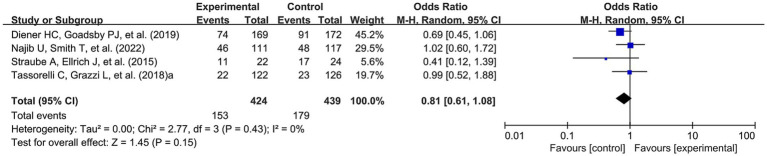
Forest plots of the effects of nVNS on safety outcomes and tolerability.

As shown in Figure 6, nVNS was generally safe and well tolerated in patients with migraine compared with sham stimulation, with no significant difference between groups (OR = 0.81, 95% CI 0.61 to 1.08, *p* = 0.15). No significant heterogeneity was observed across studies (*I*^2^ = 0.00%), indicating good consistency of the findings.

### 3.6 Subgroup analysis

Exploratory subgroup analyses were conducted according to stimulation type for the primary outcomes, including monthly headache days, monthly acute migraine medication days, and headache intensity. In the cervical nVNS subgroup, cervical stimulation was associated with significant reductions in monthly headache days (MD = −1.25, 95% CI − 1.82 to −0.68, *p* < 0.001; *I*^2^ = 4.63%) and monthly acute migraine medication days (MD = −1.02, 95% CI − 1.63 to −0.41, p < 0.001; *I*^2^ = 0.00%). Cervical nVNS also showed a significant reduction in headache intensity (MD = −1.03, 95% CI − 1.79 to −0.27, *p* = 0.01), although this estimate was derived from a single study-level effect and should therefore be interpreted cautiously (Supplementary Figures 1A–C).

In the auricular nVNS/taVNS subgroup, auricular stimulation was also associated with significant reductions in monthly headache days (MD = −1.78, 95% CI − 2.22 to −1.34, *p* < 0.001; *I*^2^ = 0.00%), monthly acute migraine medication days (MD = −1.83, 95% CI − 3.05 to −0.61, *p* = 0.003; *I*^2^ = 0.00%), and headache intensity (MD = −1.29, 95% CI − 1.80 to −0.79, *p* < 0.001; *I*^2^ = 0.00%) (Supplementary Figures 2A–C).

Overall, the direction of effect favored nVNS in both stimulation-type subgroups across the primary outcomes. However, because the number of independent trials and effect estimates within each subgroup was limited, these subgroup findings should be interpreted as exploratory and should not be considered definitive evidence of differential efficacy between cervical and auricular stimulation.

### 3.7 Sensitivity analyses

Sensitivity analyses were performed to test the robustness of the pooled results. Leave-one-out analyses for monthly headache days and monthly acute migraine medication days showed that omitting each study in turn did not meaningfully affect the direction or statistical significance of the pooled effects, although small changes in effect estimates were noted. In addition, random-effects sensitivity analyses were performed for the continuous primary outcomes to account for expected clinical heterogeneity across studies. The results remained consistent with the main analyses. Specifically, nVNS was still associated with reductions in monthly headache days (MD = −0.98, 95% CI − 1.90 to −0.16; *I*^2^ = 6.19%), monthly acute migraine medication days (MD = −0.46, 95% CI − 0.59 to −0.13; *I*^2^ = 29.42%), and headache intensity (MD = −0.92, 95% CI − 0.99 to −0.46; *I*^2^ = 0.00%). These findings suggest that the main results were robust under a random-effects model (Supplementary Figure 3).

### 3.8 GRADE evaluation of evidence

Table 2 presents the GRADE ratings and summary of findings for all pooled outcomes, including the primary continuous outcomes, acute treatment outcomes, and adverse events. The certainty of evidence was rated as high for monthly headache days and monthly acute migraine medication days, as these outcomes showed statistically significant effects, low heterogeneity, and confidence intervals that did not cross the line of no effect. The certainty of evidence for headache intensity was rated as moderate because it was downgraded for imprecision due to the small total sample size and limited number of studies.

**Table 2:** Summary of findings and GRADE certainty of evidence for all pooled outcomes.

Outcomes	No. of studies	No. of patients	Mean Difference [± 95% CI]	Anticipated absolute effects (95% CI)	*p*-value	Heterogeneity assessment	Reason for downgrading
			Grade evaluation	Risk with nVNS		I^2^ [*p*-value]	
Headache days	6	674	MD = -1.52	1.52 lower	*p* < 0.001	*I*^2^ = 23.45%	No downgrade. The total sample size was sufficient, the 95% CI did not cross the no-effect line, heterogeneity was low, and the direction of effect was consistent.
			95% CI [−1.86 to −1.18]	(1.86 lower to 1.18 lower)		[*P* = 0.24]	
			⊕ ⊕ ⊕⊕				
			High				
Acute medication days	4	550	MD = -1.18	1.18 lower	*p* < 0.001	*I*^2^ = 0.00%	No downgrade. The total sample size was >400 participants; the 95% CI did not cross the no-effect line; heterogeneity was not observed; and the direction of effect was consistent.
			95% CI [−1.72 to −0.63]	(1.72 lower to 0.63 lower)		[*P* = 0.47]	
			⊕ ⊕ ⊕⊕				
			High				
Headache intensity (VAS)	3	149	MD = -0.97	0.97 lower	*p* < 0.001	*I*^2^ = 4.41%	Downgraded one level for imprecision because the estimate was based on a small total sample size (<400 participants) and a limited number of studies.
			95% CI [−1.30 to −0.64]	(1.3 lower to 0.64 lower)		[*P* = 0.38]	
			⊕ ⊕ ⊕⊝				
			Moderate				
Pain-free within 2 h, first treated attack	2	255	OR = 2.04	Not calculated	*p* < 0.001	*I*^2^ = 0.00%	Downgraded one level for imprecision because the estimate was based on only two studies with a limited sample size and event data.
			95% CI [1.42 to 2.95]			[*P* = 0.58]	
			⊕ ⊕ ⊕⊝				
			Moderate				
Pain-free within 2 h, all attacks	2	255	OR = 1.82	Not calculated	*p* < 0.001	*I*^2^ = 0.00%	Downgraded one level for imprecision because the estimate was based on only two studies with a limited sample size and event data.
			95% CI [1.43 to 2.31]			[*P* = 0.89]	
			⊕ ⊕ ⊕⊝				
			Moderate				
Pain relief within 2 h, first treated attack	2	255	OR = 1.85	Not calculated	*p* < 0.001	*I*^2^ = 0.00%	Downgraded one level for imprecision because the estimate was based on only two studies with a limited sample size and event data.
			95% CI [1.47 to 2.33]			[*P* = 0.98]	
			⊕ ⊕ ⊕⊝				
			Moderate				
Pain relief within 2 h, all attacks	2	255	OR = 1.68	Not calculated	*p* < 0.001	*I*^2^ = 33.00%	Downgraded one level for imprecision because the estimate was based on only two studies with a limited sample size and event data.
			95% CI [1.45 to 1.94]			[P = 0.19]	
			⊕ ⊕ ⊕⊝				
			Moderate				
Adverse events	4	734	OR = 0.81	Not calculated	*p* = 0.15	*I*^2^ = 0.00%	Downgraded one level for imprecision because the 95% CI crossed the line of no effect and the estimate was based on limited adverse-event data.
			95% CI [0.61 to 1.08]			[*p* = 0.43]	
			⊕ ⊕ ⊕⊝				
			Moderate				

GRADE: Grading of Recommendations, Assessment, Development, and Evaluations; nVNS: Non-invasive vagus nerve stimulation; MD: Mean Difference; OR: odds ratio. Imprecision was judged based on the number of included studies, total sample size or event data, and the width and clinical relevance of the 95% CI. Outcomes were not downgraded solely on sample size when the confidence interval was narrow, did not cross the line of no effect, and the direction of effect was consistent.

For the acute treatment outcomes, including pain-free and pain-relief responses within 2 h after the first treated attack and across all treated attacks, the certainty of evidence was rated as moderate. These outcomes were downgraded for imprecision because the estimates were based on only two studies with a limited sample size and event data. The certainty of evidence for adverse events was also rated as moderate, as the 95% CI crossed the line of no effect and the estimate was based on limited adverse-event data. Detailed GRADE judgments and reasons for downgrading are presented in Table 2.

## 4 Discussion

This systematic review and meta-analysis included nine publications corresponding to seven independent RCTs involving 917 unique participants and comprehensively evaluated the efficacy and safety of nVNS for migraine management. nVNS was associated with reductions in monthly headache days, monthly acute migraine medication days, and headache pain intensity. Exploratory subgroup analyses by stimulation type showed favorable effects in both cervical nVNS and auricular nVNS/taVNS subgroups, although these findings should be interpreted cautiously because of the limited number of trials. For acute treatment outcomes, nVNS also showed favorable effects on pain-free and pain-relief responses within 2 h after treatment. These outcomes were stratified by post-treatment assessment time point, and time-point-specific subgroup findings were prioritized. The exploratory overall estimates across time points were consistent with the subgroup findings but should be interpreted cautiously because repeated time-point data may be statistically correlated. Overall heterogeneity was low, sensitivity analyses supported result stability, and safety analyses indicated that nVNS was generally well tolerated. GRADE assessment showed high certainty for monthly headache days and monthly acute migraine medication days, and moderate certainty for headache intensity, acute treatment outcomes, and adverse events. Together, these findings suggest that nVNS may be a promising non-pharmacological option for both acute and preventive migraine management.

Although previous individual studies have not reached fully consistent conclusions regarding the efficacy of nVNS, the pooled findings of this review generally support its potential clinical value in migraine management (13, 19). In the present analysis, nVNS was associated with reductions in monthly headache days, monthly acute migraine medication days, and headache pain intensity. For acute treatment outcomes, nVNS also showed favorable effects on pain-free and pain-relief responses within 2 h after treatment, both after the first treated attack and across all treated attacks. Because several trials reported these acute outcomes at multiple post-treatment time points, the analyses were stratified by assessment time point, and the time-point-specific subgroup findings were prioritized when interpreting treatment effects. The exploratory overall estimates across time points were generally consistent with these findings. Still, they should be interpreted cautiously because repeated time-point data from the same trial population may be statistically correlated.

The reduction in monthly acute medication days may be particularly clinically relevant, as it reflects not only a lower headache burden but also a potential reduction in reliance on acute medications, which may help decrease the risks of adverse drug effects, drug interactions, and medication overuse. Nevertheless, these findings should still be considered in the context of inconsistent results from earlier studies. For example, the EVENT study did not demonstrate a significant improvement in headache days in patients with chronic migraine, while the PREMIUM study failed to show a significant between-group difference in monthly migraine days in the intention-to-treat analysis of episodic migraine. In contrast, some taVNS studies suggested reductions in headache days and pain intensity (12, 13, 19). These discrepancies indicate that the effects of nVNS may not be uniform across all migraine subtypes or study settings, and may depend on factors such as stimulation site, stimulation parameters, timing of intervention, treatment duration, and patient characteristics (22, 23). From a clinical perspective, the value of nVNS may therefore extend beyond improvement in any single outcome, as it offers a repeatable, relatively well-tolerated, and potentially earlier-to-initiate treatment option without increasing medication-related risks. Particularly in the early stage of an acute attack, timely initiation of nVNS while pain remains mild may help prevent attack progression and reduce the subsequent need for rescue medication. Accordingly, its clinical relevance may be better understood as an optimization of the overall migraine management pathway rather than merely an improvement in selected statistical endpoints.

Exploratory subgroup analyses by stimulation type were performed to address the clinical heterogeneity between cervical and auricular stimulation. The results showed that both cervical nVNS and auricular nVNS/taVNS were associated with favorable effects on the primary outcomes, including monthly headache days, monthly acute migraine medication days, and headache intensity. This suggests that the overall beneficial direction of nVNS was not driven solely by one stimulation modality. However, cervical and auricular stimulation differ in anatomical targets, stimulation parameters, devices, treatment schedules, and clinical application (24). Moreover, because the number of independent trials and effect estimates within each subgroup was limited, these findings should be interpreted as exploratory and should not be considered definitive evidence that the two stimulation modalities have equivalent efficacy.

The time-point-specific subgroup analyses provide additional insight into the temporal pattern and clinical relevance of nVNS effects. For preventive outcomes, reductions in monthly headache days were generally consistent across 4, 8, and 12 weeks, although the 8-week estimate was not statistically significant, likely due to the limited number of studies and wide confidence interval. Headache intensity showed relatively stable improvement across time points, whereas the time course of acute medication use was less consistent. From the perspective of clinical relevance, the pooled reduction in monthly headache days was approximately 1.5 days per month, which reaches or slightly exceeds the commonly cited MCID range for migraine of approximately 1.0–1.5 headache days per month. This suggests that the preventive effect of nVNS on monthly headache days may be clinically meaningful, rather than only statistically significant. However, because a universally accepted MCID specifically for nVNS-treated migraine populations has not been established, this interpretation should remain cautious and should consider baseline headache frequency, migraine subtype, treatment context, and patient expectations. For acute treatment, previous PRESTO findings support an early analgesic effect of nVNS, with benefits observed as early as 30 min and up to 60 min after treatment, and further analyses showing favorable pain-free and pain-relief responses at 60 and 120 min across treated attacks (11). These findings are broadly consistent with our subgroup results, in which pain-relief responses within 2 h generally favored nVNS across different assessment time points. However, some pain-free subgroup estimates did not reach statistical significance, probably because of limited statistical power. Therefore, the apparent time-dependent pattern should be interpreted cautiously, and future trials with standardized assessment time points are needed to clarify the onset, duration, and clinical importance of nVNS effects.

These clinical and subgroup findings may be biologically plausible given the multifaceted modulatory effects of nVNS on migraine-related neurophysiological processes. Although the exact mechanisms by which different forms of nVNS exert therapeutic effects in migraine remain incompletely understood, existing basic and animal studies provide plausible biological support. First, vagus nerve stimulation has been shown to inhibit cortical spreading depression (CSD), which is considered one of the key pathophysiological processes underlying migraine attacks, particularly migraine with aura (25, 26). Second, nVNS may exert analgesic effects by modulating the imbalance between excitatory and inhibitory neurotransmission, for example, by enhancing GABA- and serotonin-related descending inhibitory pathways while reducing levels of excitatory neurotransmitters such as glutamate, thereby potentially suppressing excessive activation of the trigeminal system (27–29). In addition, nVNS may influence the expression of proteins associated with central and peripheral sensitization, thereby attenuating trigeminal neuronal activation and subsequent amplification of pain signaling (30, 31). Notably, previous studies have suggested that earlier initiation of nVNS may be more effective in suppressing neurotransmitter release and rising central excitability before severe pain becomes established, which may partly explain its potential value when used early during an acute attack (32, 33). This mechanism-based interpretation is consistent with the time-point-specific subgroup findings, which suggested favorable pain-relief responses within the early post-treatment period. Nevertheless, because cervical nVNS and auricular nVNS/taVNS stimulate different anatomical regions and may engage vagal afferent pathways in different ways, their specific neurophysiological effects may not be fully interchangeable. Taken together, nVNS may alleviate migraine through multiple mechanisms, including inhibition of CSD, modulation of neurotransmitter balance, suppression of trigeminal activation, and reduction of central sensitization.

Compared with previous systematic reviews and meta-analyses, the present study offers several important updates and added value (14, 34). First, we included a larger and more recent body of evidence, comprising nine publications corresponding to seven independent RCTs, and applied no language restrictions during the literature search, which broadened the evidence base and may have reduced the risk of language bias. Second, this review provided a more comprehensive evaluation of different forms of non-invasive vagus nerve stimulation, including both non-invasive cervical and auricular approaches, as well as different stimulation patterns, thereby offering a broader perspective on the potential role of nVNS in migraine treatment. Third, given that nVNS may be used for both acute treatment and preventive management, we incorporated outcomes relevant to both settings. In addition to short-term outcomes such as pain-free and pain-relief responses within 2 h, which were further stratified by post-treatment assessment time point, we also synthesized key burden-related and preventive outcomes, including monthly headache days, monthly acute migraine medication days, and headache pain intensity. Moreover, by stratifying acute treatment outcomes according to post-treatment assessment time points, this review provided a more detailed assessment of the temporal profile of nVNS effects, which may help clarify whether the therapeutic response emerges early and persists during the acute attack period. Therefore, compared with earlier reviews that mainly focused on short-term pain relief or a limited range of outcomes, the present study provides a more comprehensive assessment of the potential role of nVNS across different migraine management settings.

In terms of safety, the present findings are in line with earlier systematic reviews and meta-analyses on taVNS, which have generally indicated that nVNS is safe and well tolerated. Previous studies have shown that active taVNS does not significantly increase adverse events compared with control conditions, and no clearly stimulation-related serious adverse events have been identified (35). Reported side effects were usually mild and transient, most commonly ear pain, headache, tingling, and local skin irritation. Similarly, prior reviews of nVNS in migraine have also suggested a favorable overall tolerability profile. Consistent with this evidence, our analysis found no significant difference in adverse events between nVNS and sham stimulation, further supporting its clinical acceptability in patients with migraine. In addition, the GRADE assessment showed that the certainty of evidence for the primary outcomes was moderate to high, which increases confidence in the main efficacy findings. However, given the limited number of available studies and the incomplete reporting of adverse events in some trials, further large-scale studies with longer follow-up are still needed to clarify the long-term safety of nVNS and possible differences among stimulation modalities.

## 5 Limitation

Despite providing a relatively comprehensive evaluation of the efficacy and safety of nVNS in migraine management, this study has several limitations. First, some outcomes were based on a small number of RCTs and participants, which may have limited the precision of the pooled estimates. Second, although subgroup analyses by stimulation type were performed, cervical nVNS and auricular nVNS/taVNS differ in stimulation anatomy, device characteristics, stimulation parameters, treatment schedules, and clinical intent. The limited number of trials within each subgroup precluded firm conclusions regarding whether treatment effects differ between these stimulation approaches. Third, the included studies covered both preventive treatment trials and acute-attack treatment trials, which differed in eligibility criteria, treatment duration, timing of outcome assessment, and clinical endpoints. Therefore, this design-level heterogeneity should be considered when interpreting the pooled findings. Fourth, several outcomes were reported at multiple assessment time points, and exploratory overall estimates across time points should be interpreted cautiously because repeated time-point data from the same trial population may be statistically correlated. Fifth, adverse event reporting was incomplete in some trials, and follow-up periods were relatively short, leaving the long-term safety of nVNS uncertain. Finally, because fewer than 10 independent RCTs were available for each outcome, publication bias was not formally assessed. Further large, multicenter RCTs with standardized stimulation protocols, clearly defined treatment intent, consistent outcome assessment time points, and longer follow-up are needed.

## 6 Conclusion

This study suggests that nVNS may improve several important clinical outcomes in patients with migraine, including reductions in monthly headache days, monthly acute migraine medication days, and headache pain intensity, as well as favorable pain-free and pain-relief responses within 2 h during acute attacks. nVNS also appears to have a favorable overall safety and tolerability profile. Exploratory subgroup analyses suggested favorable effects for both cervical nVNS and auricular nVNS/taVNS, but these findings should be interpreted cautiously because of the limited number of trials and clinical heterogeneity across stimulation modalities. Overall, these findings indicate that nVNS may have potential clinical value in both acute treatment and preventive management of migraine. However, further high-quality studies are needed to clarify the optimal stimulation strategy, long-term safety, and the patient groups most likely to benefit.

## Data Availability

The original contributions presented in the study are included in the article/Supplementary material, further inquiries can be directed to the corresponding author/s.

## Supplementary material

SUPPLEMENTARY FIGURE 1 Exploratory subgroup analyses of cervical nVNS for primary outcomes. **(A)** Monthly headache days; **(B)** monthly acute migraine medication days; **(C)** headache intensity.

SUPPLEMENTARY FIGURE 2 Exploratory subgroup analyses of auricular aVNS for primary outcomes. **(A)** Monthly headache days; **(B)** monthly acute migraine medication days; **(C)** headache intensity.

SUPPLEMENTARY FIGURE 3 Random-effects sensitivity analyses for continuous primary outcomes. **(A)** Monthly headache days; **(B)** monthly acute migraine medication days; **(C)** headache intensity.

SUPPLEMENTARY TABLE 1 Search strategy.

SUPPLEMENTARY TABLE 2 Raw data.
