# Supplementary material for: The effectiveness of non-invasive vagus nerve stimulation in migraine management: a meta-analysis of randomized controlled trials
Source: Front Neurol. 2026 Jul 27;17:1842386. doi: 10.3389/fneur.2026.1842386 (PMC13456144; doi:10.3389/fneur.2026.1842386)
Supplement: Supplementary file 1 [file Table_1.DOCX]

**Supplementary Table 1.** Search Strategy.

#1 headache disorders [Mesh]

#2 headache [Mesh]

#3 (headache* OR migrain* OR cephalgi* OR cephalalgi* OR hemicrani*) [Title/Abstract]

#4 #1 OR #2 OR #3

#5 Vagus Nerve [Mesh]

#6 ((vagus OR vagal) And stimul*) [Title/Abstract]

#7 #5 OR #6

#8 (randomized controlled trial[pt] OR controlled clinical trial[pt] OR randomized[tiab] OR placebo[tiab] OR clinical trials as topic[mesh:noexp] OR randomly[tiab] OR trial[ti]) NOT (animals [mh] NOT (humans [mh] AND animals[mh]))

#9 #4 And #7 And #8
